# Effect of nursing intervention to guide early postoperative activities on rapid rehabilitation of patients undergoing abdominal surgery: A protocol for systematic review and meta-analysis

**DOI:** 10.1097/MD.0000000000024776

**Published:** 2021-03-26

**Authors:** Lu Rao, Xinjian Liu, Li Yu, Hui Xiao

**Affiliations:** a Guangzhou University of Chinese Medicine, Guangzhou; b Shenzhen Nanshan District Shekou People's Hospital, Shenzhen; c The Second Affiliated Hospital of Guangzhou University of Chinese Medicine, Guangzhou, Guangdong Province, China.

**Keywords:** abdominal surgery, early postoperative activities, enhanced recovery after surgery, protocol, refined nursing, systematic review

## Abstract

**Background:**

Postoperative complications after abdominal surgery are high, and there is no reliable intervention program to prevent them. Some studies have pointed out that early postoperative activities have advantages in preventing the occurrence of complications, but lack of evidence-based basis. The purpose of this study is to systematically evaluate the effect of nursing intervention is guiding early postoperative activities on the rapid recovery of patients undergoing abdominal surgery.

**Methods:**

China National Knowledge Infrastructure, Wanfang, China Science and Technology Journal Database and Chinese Biomedical Database, PubMed, Embase, Web of Science and the Cochrane Library will be searched by computer, and a randomized controlled study is conducted on early participation in exercise programs after abdominal surgery from the establishment of the database to January 2021. The language is limited to English and Chinese. The quality of the included study is independently extracted and the literature quality is evaluated by 2 researchers, and the included literature is analyzed by Meta using RevMan5.3 software.

**Results:**

This study will evaluate the effect of nursing intervention is guiding early postoperative activities on the rapid rehabilitation of patients undergoing abdominal surgery through the indexes of postoperative quality of life score, the incidence of complications, mortality, length of stay and so on.

**Conclusion:**

This study will provide reliable evidence-based basis for establishing a reasonable and effective postoperative activity guidance program for patients undergoing abdominal surgery.

**Ethics and dissemination:**

Private information from individuals will not be published. This systematic review also does not involve endangering participant rights. Ethical approval will not be required. The results may be published in a peer-reviewed journal or disseminated at relevant conferences.

**OSF Registration number:**

DOI 10.17605/OSF.IO/59MD4

## 1 Introduction

Abdominal surgery refers to the operation of abdominal organs to repair accidental injuries, peritonitis, abdominal abscess and digestive tract diseases.^[1]^ The incidence of complications after abdominal surgery is high, including pulmonary infection, abdominal distension, intestinal adhesion, venous thrombosis and so on. Among them, the incidence of intestinal adhesion is as high as 95%, and the case fatality rate is 8%-13%, which has become a major clinical challenge for abdominal surgery.^[2]^ In upper abdominal surgery, cardiopulmonary adverse events accounted for 50% of postoperative complications.^[3]^ In emergency abdominal surgery, the incidence of pulmonary complications is 20% to 50%.^[4]^ According to statistics, more than 20% of patients undergoing abdominal surgery have postoperative complications that require invasive treatment, which greatly increases the risk of further morbidity and death.^[5]^ Although clinicians continue to actively explore intervention measures, such as postoperative “enhanced postoperative recovery” program, to reduce postoperative complications,^[6]^ there is still no reliable solution to solve this problem.

In recent years, the concept of enhanced recovery after surgery is constantly developing and improving, and the rapid recovery of patients undergoing abdominal surgery is also an important part. Among a series of strategies for rapid rehabilitation, getting out-of-bed early after operation is an important part of it.^[7]^ It is pointed out that early getting out of bed after an operation can promote the recovery of gastrointestinal function and prevent abdominal distension. At the same time, it can effectively avoid complications such as venous thrombosis of the lower extremities and pulmonary infection.^[8]^ Guiding early out-of-bed activities of patients undergoing abdominal surgery has become one of the basic contents of nursing.^[9]^ Chen et al^[10]^ found in the study of 179 elderly patients after abdominal surgery that nursing intervention, including early exercise and therapeutic exercise, can reduce the decline of physical function and the incidence of delirium in elderly patients after abdominal surgery. Basse et al^[11]^ found in their study that early exercise and rapid rehabilitation intervention can prevent the decline of pulmonary function after enterectomy and the decline of cardiopulmonary function during exercise.

Although a number of randomized controlled studies have confirmed that early postoperative getting out of bed activity is of positive significance for patients’ rapid rehabilitation after operation,^[8,12–15]^ the results are different because of the difference in research scheme and nursing content. At the same time, the timing and time of getting out-of-bed early after operation are not uniform, lack of reliable evidence-based basis, which limits the promotion of this program. Therefore, this study plans to systematically evaluate the effect of nursing intervention is guiding early postoperative activities on the rapid rehabilitation of patients undergoing abdominal surgery, in order to get a standardized and reliable conclusion.

## 2 Methods

### 2.1 Protocol register

This protocol of systematic review and meta-analysis has been drafted under the guidance of the preferred reporting items for systematic reviews and meta-analyses protocols. Moreover, it has been registered on open science framework (OSF) (Registration number: DOI 10.17605/OSF.IO/59MD4).

### 2.2 Ethics

Since the program does not require the recruitment of patients and the collection of personal information, it does not require the approval of the Ethics Committee.

### 2.3 Inclusion criteria

The randomized controlled trials study of all patients undergoing abdominal surgery with different indications and the comparison of the results of early postoperative activity-directed nursing and standard nursing without activity plan is in line with the conditions for inclusion in the evaluation of this system.

### 2.4 Exclusion criteria

(1) The published literature is abstract, review and animal research.
(2) Repeatedly published literature for the same research population.
(3) Articles with incomplete or incorrect data and unable to obtain complete data after contacting the author.
(4) The literature with inconsistent intervention methods or no related outcome indicators.

### 2.5 Outcome index

Main outcome measures: ① postoperative quality of life evaluation, including: 6 min walking test distance, short form 36 questionnaire score (the MOS item short from health survey) system, ② postoperative complications, including: pulmonary infection, abdominal distension, intestinal adhesion, venous thrombosis of lower extremities, and so on.

Secondary outcome measures: length of hospital stay; mortality; incidence of adverse reactions (adverse events related to early activities, such as falls, etc.).

### 2.6 Retrieval strategy

The Chinese words “surgery,” “abdominal operation,” “tumor,” “early exercise,” “postoperative activity” and“Nursing” are searched in China National Knowledge Infrastructure, Wanfang Data Knowledge Service Platform, China Science and Technology Journal Database, Chinese Journal Service platform, and China Biomedical Database. The English words “Abdominal Surgery,” “Abdominal Operation,” “Early Ambulation,” “Early postoperative activities” and “Nursing” are searched in PubMed, EMBASE, Web of Science and the Cochrane Library. The retrieval time is of the establishment of the database in January 2021, and all the domestic and foreign literature about early participation in exercise programs after abdominal surgery will be collected. Take PubMed as an example, the retrieval strategy is shown in Table 1.

**Table 1:** Search strategy in PubMed database.

Number	Search terms
#1	early ambulation [Title/Abstract]
#2	postoperative activities [Title/Abstract]
#3	postoperative training [Title/Abstract]
#4	postoperative exercise [Title/Abstract]
#5	postoperative physical therapy [Title/Abstract]
#6	postoperative sport [Title/Abstract]
#7	postoperative rehabilitation [Title/Abstract]
#8	Nursing [Title/Abstract]
#9	#1 OR #2 OR #3 OR #4 OR #5 OR #6 OR #7 OR #8
#10	Neoplasms [MeSH]
#11	surgery [Subheading]
#12	cancer [Title/Abstract]
#13	tumor [Title/Abstract]
#14	Malignant Neoplasm [Title/Abstract]
#15	abdominal surgery [Title/Abstract]
#16	abdominal operation [Title/Abstract]
#17	abdomen surgery [Title/Abstract]
#18	abdomen operation [Title/Abstract]
#19	#10 OR #11 OR #12 OR #13 OR #14 OR #15 OR #16 OR #17 OR #18
#20	#9 AND #19

### 2.7 Data screening and extraction

**Figure 1:**
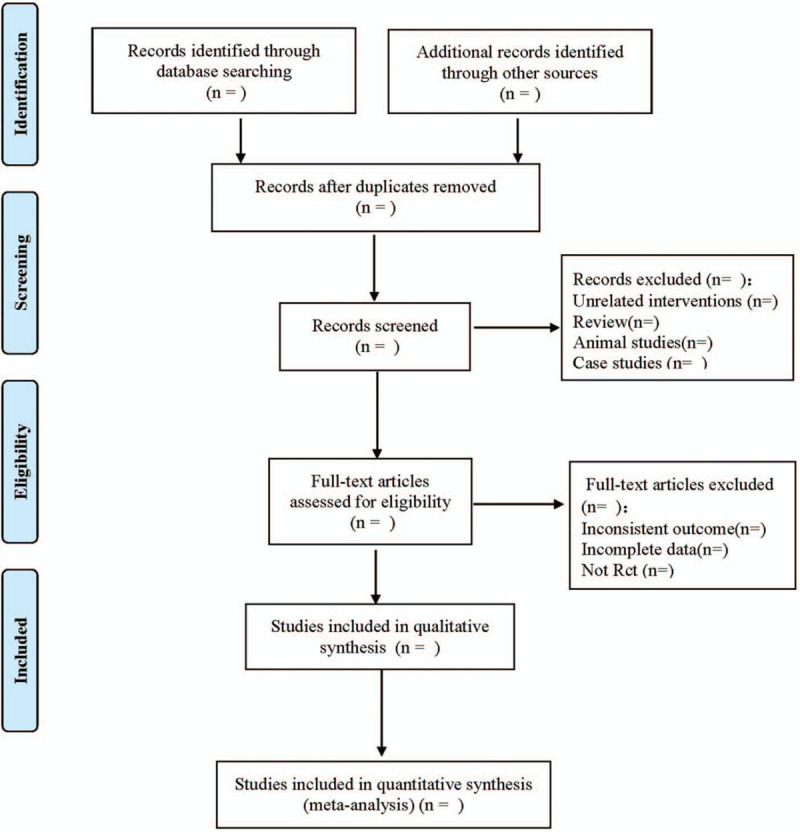
Flow diagram.

The data will be extracted independently by the two researchers, the information will be recorded in the data extraction form, and their differences will be resolved with the help of a third reviewer. The detailed information is as follows: ① Clinical study (title, first author, publication date, sample size, sex ratio, average age, length of stay, type of operation); ② intervention measures (treatment group and control group nursing type, treatment group exercise plan, duration, follow-up time); ③ Randomized controlled trials; ④ outcome indicators. The literature screening process is shown in Figure 1.

### 2.8 Literature quality assessment

The quality evaluation of the literature is completed independently by two researchers according to the Cochrane5.1.0 system evaluation manual. And in case of disagreement, the third researcher participates in the discussion to finally decide the overall quality of the literature. Evaluation indicators include random sequence generation, distribution hiding, blind method, integrity of result data, selective reporting of research results and other sources of bias. According to these indicators, the included literature is evaluated as “high bias risk”, “low bias risk” and “unknown.”

### 2.9 Statistical analysis

#### 2.9.1 Data analysis and processing

RevMan 5. 3 software is used for meta-analysis. X^2^ tests and *I*^*2*^ are used to determine whether there is heterogeneity in each literature, and the heterogeneity of the effect value is analyzed. If *P* *>* .1, *I*^*2*^ *<* 50%, it shows that the heterogeneity among the studies is low, so a fixed model is used for analysis; if *P* *<* .1*, I*^*2*^* ≥* 50%, there is obvious heterogeneity among the studies. The sources of heterogeneity are analyzed and the random effect model is used for analysis. The measurement data are expressed as the weighted mean difference or the standard mean difference and 95% confidence interval, and the counting data are expressed as relative ratio and 95% confidence interval.

#### 2.9.2 Dealing with missing data

If the data of the required study are incomplete or not reported in the study, the researcher will contact the first author or other author of the study by phone or email. If the required data are not available, we will use descriptive analysis instead of meta- analysis and exclude these studies if necessary.

#### 2.9.3 Subgroup analysis

We will further explore the effects of different exercise programs on the results of different abdominal surgery patients according to the type of operation, postoperative exercise time, type of postoperative exercise, patient age, and other factors.

#### 2.9.4 Sensitivity analysis

According to the recommendations of the Cochrane manual, we will conduct a sensitivity analysis of each outcome index. In order to test the stability of meta-analysis results of indicators, an 1-by-1 elimination method will be adopted for sensitivity analysis.

#### 2.9.5 Assessment of reporting biases

If there are more than 10 studies, funnel chart is used to evaluate whether there is publication bias. Moreover, Egger and Begg test are used for the evaluation of potential publication bias.

#### 2.9.6 Evidence quality evaluation

We will use the Grading of Recommendation Assessment, Development and Evaluation scoring method to grade the evidence of the outcome index.^[16]^ The evaluation content includes bias risk, indirectness, inconsistency, inaccuracy and publication bias, and the quality of evidence will be rated as high, medium, low or very low.

## 3 Discussion

According to the traditional view, it is necessary to stay in bed after the operation to reduce the body's oxygen consumption, relieve pain and promote wound healing. However, a series of studies have found that postoperative bed rest has many harms, such as decreased insulin sensitivity, atelectasis, decreased exercise ability, muscle atrophy, bone loss, thrombotic disease, microvascular dysfunction, stress injury and so on.^[17,18]^ Moreover, the gastrointestinal function of patients undergoing abdominal surgery weakens or disappears due to stimulation such as anesthesia, surgical trauma and traction, which is manifested abdominal pain, abdominal distension, dysfunction of exhaust and defecation, etc. If intestinal peristalsis is not restored as soon as possible, it will lead to the accumulation of toxins in the body, which seriously affects the recovery of the patient's body and the prognosis of the disease.^[19]^ Therefore, the importance of getting out of bed early after the operation is self-evident and widely respected. For example, many guidelines issued by enhanced recovery after surgery Society and the consensus of relevant domestic experts all list it as “highly recommended”.^[20,21]^ Clinical studies have confirmed that early postoperative exercise can maintain the normal tension of the muscles of the whole body, promote the metabolism and blood circulation of various systems of the body, and promote the regeneration, repair and functional remodeling of tissue injury.^[22]^ Reduce postoperative complications such as pulmonary complications and venous thrombosis of the lower extremities;^[23,24]^ increase physical coordination and self-care ability.^[25]^

Because of incision pain, worry about incision rupture, postoperative fatigue, upright intolerance, postoperative anxiety and other reasons, patients refuse to get out of bed or delay the time of getting out of bed,^[26,27]^ and most patients do not know how to exercise early, do not know how to exercise, do not know how to arrange the amount of activity,^[26]^ standardize nursing guidance and intervention after operation plays a decisive role in whether patients can effectively achieve positive impact. Through this systematic evaluation, we will provide a reliable evidence-based basis for the establishment of a standardized and effective postoperative activity guidance program for patients undergoing abdominal surgery.

However, this systematic review has some limitations. Due to the different types of surgery, the time of getting out of bed, exercise intensity, type and other factors will increase the possibility of heterogeneity. Due to the limitations of language retrieval, we will only include Chinese and English literature, and studies in other languages and regions may be ignored.

## Author contributions

**Data collection**: Lu Rao and Xinjian Liu

**Funding support**: Lu Rao

**Resources**: Xinjian Liu and Li Yu

**Software operating**: Li Yu and Hui Xiao

**Supervision**: Lu Rao

**Writing – original draft**: Lu Rao and Xinjian Liu

**Writing – review & editing**: Lu Rao
